# Older age and depressive state are risk factors for re-positivity with SARS-CoV-2 Omicron variant

**DOI:** 10.3389/fpubh.2022.1014470

**Published:** 2022-10-04

**Authors:** Maojun Li, Huawen Peng, Guangyou Duan, Jian Wang, Zhiqing Yu, Zhongrong Zhang, Liping Wu, Ming Du, Shiji Zhou

**Affiliations:** ^1^Department of Anesthesiology, People‘s Hospital of Linshui County, Guangan, China; ^2^People‘s Hospital of Linshui County, Guangan, China; ^3^Department of Anesthesiology, The Second Affiliated Hospital of Chongqing Medical University, Chongqing, China; ^4^Department of General Surgery, People‘s Hospital of Linshui County, Guangan, China; ^5^Traditional Chinese Medicine Hospital of Linshui County, Guangan, China; ^6^Department of Gastrointestinal Surgery, The Second Affiliated Hospital of Chongqing Medical University, Chongqing, China

**Keywords:** Omicron, reinfection, COVID-19, depression, anxiety, sleep, older adults, SARS-CoV-2

## Abstract

**Background:**

The reinfection rate of SARS-CoV-2 Omicron variant is high; thus, exploring the risk factors for reinfection is important for the effective control of the epidemic. This study aimed to explore the effects of psychological and sleep factors on re-positivity with Omicron.

**Methods:**

Through a prospective cohort study, 933 adult patients diagnosed with Omicron BA.2.2 infection and testing negative after treatment were included for screening and follow-up. We collected data on patients' demographic characteristics, SARS-CoV-2 Omicron vaccination status, anxiety, depression, and sleep status. Patients underwent nucleic acid testing for SARS-CoV-2 Omicron for 30 days. Regression and Kaplan-Meier analyses were used to determine the risk factors for re-positivity of Omicron.

**Results:**

Ultimately, 683 patients were included in the analysis. Logistic regression analysis showed that older age (*P* = 0.006) and depressive status (*P* = 0.006) were two independent risk factors for Omicron re-positivity. The odds ratios of re-positivity in patients aged ≥60 years and with a Patient Health Questionnaire-9 (PHQ-9) score ≥5 was 1.82 (95% confidence interval:1.18–2.78) and 2.22 (1.27–3.85), respectively. In addition, the time from infection to recovery was significantly longer in patients aged ≥60 years (17.2 ± 4.5 *vs*. 16.0 ± 4.4, *P* = 0.003) and in patients with PHQ-9≥5 (17.5 ± 4.2*vs*. 16.2 ± 4.5, *P* = 0.026). Kaplan–Meier analysis showed that there was a significantly higher primary re-positivity rate in patients aged ≥60 years (*P* = 0.004) and PHQ-9 ≥ 5 (*P* = 0.007).

**Conclusion:**

This study demonstrated that age of ≥60 years and depressive status were two independent risk factors for re-positivity with Omicron and that these factors could prolong the time from infection to recovery. Thus, it is necessary to pay particular attention to older adults and patients in a depressive state.

## Introduction

On November 26, 2021, the WHO declared a new SARS-CoV-2 variant strain named Omicron (B.1.1529 variant) as a variant of concern (1). On May 9, 2022, in a remote county in Sichuan Province, China, with a resident population of ~600,000, a sudden Omicron epidemic proliferated across the entire county, including rural areas. Through the gene sequencing, the variant of Omicron was determined as BA.2. 2 by Sichuan Center for Disease Control and Prevention. There were 1,268 confirmed infections, including in 933 adults. Existing studies have shown that the Omicron variant involves new mutations in its spike protein, most of which are located at its receptor-binding site. These changes make it more infectious and transmissible, and simultaneously cause immune escape. This leads to reduced efficacy and poor therapeutic effect of existing vaccines (2, 3), and it usually spreads widely before symptoms appear (4).

Some studies indicate that there are still many Omicron infections in South Africa, despite comprehensive vaccination and 90% group immunization, demonstrating that Omicron is highly contagious and allows immune escape to vaccines and antibodies (5). Although incidences of severe illness and mortality induced by Omicron were lower than previous variants, it was more transmissible and less well-controlled by vaccination (6, 7). The high infectivity made the Omicron threaten more people, especially for elderly (8, 9), and one study have demonstrated that rate of severe illness would not be decreased for some population without vaccination (10). Experimental studies show that Omicron has many mutations in its structure compared with SARS-CoV-2, which increases the risk of reinfection (2, 11–13). In previous outbreaks, the total reinfection rate of the SARS-CoV-2 variant was <2% (6). Before Omicron emerged, the low reinfection rate was due to the production of immune antibodies after vaccination and infection, and the protective efficacy was greatly reduced before Omicron (14). Some institutions estimate that the actual infection rate is higher (15), and compared with the Delta variant, the risk of reinfection with Omicron increases nearly 18 times (16). Considering such a high rate of infectivity and reinfection, if positive patients and susceptible populations such as older adults are not controlled in advance, Omicron will proliferate and over-burden the medical system, resulting in an increased number of deaths (8, 10). Therefore, it is necessary to identify risk factors for patients' re-positivity. If we can predict which groups are more likely to relapse, it will provide guidance for the prevention, diagnosis, and treatment of Omicron, as well as for the formulation of prevention and control strategies.

Existing studies have clarified that HIV, obesity, pregnancy, and medical workers over 60 years of age may increase the risk of reinfection with Omicron (16). However, there are very few studies on the influence of anxiety, depression, and sleep status on reinfection. Previous studies have mainly focused on physical and psychological changes after COVID-19, and it is clear that the incidence of these factors is high (17, 18). Therefore, it is essential to clarify whether these are important factors that aggravate re-positivity with Omicron. In addition, previous studies have focused on almost all large- and medium-sized cities with concentrated and developed medical resources. However, in small counties in China, people's cognition, and knowledge levels regarding the virus are seriously lacking, their panic is more obvious, and their psychological problems may be more prominent (19, 20). Therefore, this study included patients with Omicron and explored the predictability of anxiety, depression, and sleep status in Omicron re-positivity.

## Methods

### Patients

**Figure 1:**
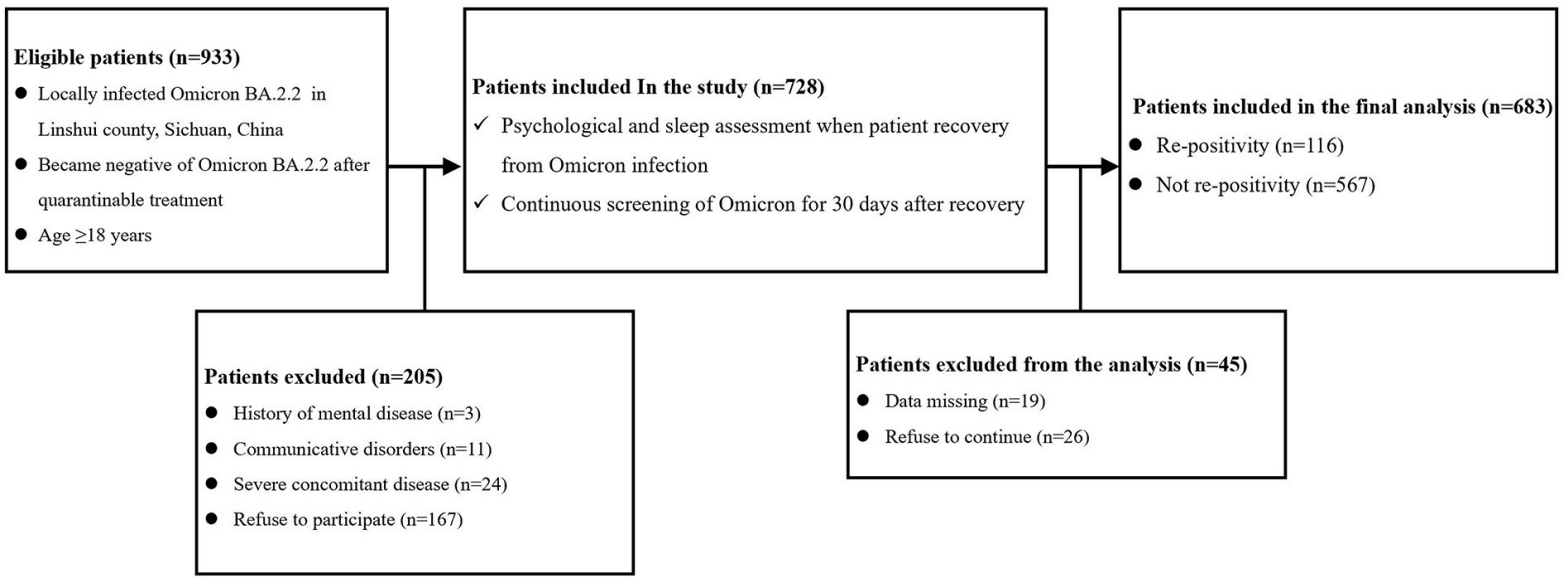
Flow chart of the study. (Complication means diseases of different systems [including the cardiovascular, respiratory, urinary, digestive, nervous, and hematological systems, etc.]; Severe concomitant disease means accompanying severe disease requiring hospitalization).

This study was a prospective cohort study. The study protocol was reviewed and approved by the ethics committee of the People's Hospital of Linshui County, Sichuan Province, China. As shown in Figure 1, a total of 933 patients who were locally diagnosed with Omicron BA.2.2 and tested negative after treatment were screened and followed. The inclusion criteria were age ≥18 years, locally diagnosed with Omicron BA.2.2, living in Linshui County, Sichuan Province, China, and testing negative for Omicron BA.2.2, after quarantinable treatment. Patients were excluded if they met any of the following criteria: diagnosed mental disease before infection, accompanying severe disease requiring hospitalization, and communicative disorders or refusal to participate.

### Study design and intervention

Patients were invited to participate in the study when they were sent to the People's Hospital of Linshui County after testing negative for Omicron BA.2.2 during quarantinable treatment. Negative diagnosis of Omicron BA.2.2 was determined by negative results for two successive tests at intervals of more than 24 h. Demographic data (sex, age, height, weight, BMI, smoking status, and drinking status) were collected. Complications of different systems (including the cardiovascular, respiratory, urinary, digestive, nervous, and hematological systems, etc.) were recorded according to the patients' chief complaints. Information regarding COVID-19 vaccination was also collected. After recording the above information, the patient was asked to complete a psychological and sleep evaluation. Nucleic acid testing for COVID-19 was performed daily for 30 days. If a patient was screened as positive for infection with Omicron BA.2.2 again, they were defined as a re-positivity case.

### Psychological and sleep evaluation

The same group of professional psychologists completed the psychological and sleep assessments. The Patient Health Questionnaire 9 (PHQ-9) was used to screen and evaluate depressive symptoms, with a total of nine items and a scale of 0 (not at all) to 3 (almost every day) (21). The total score of the PHQ-9 scale ranges from 0 to 27 points, of which 0 to 5 points indicate no depression, and a score >5 indicates a depressive state; the higher the score, the more severe the depressive symptoms. The Generalized Anxiety Disorder Scale 7 (GAD-7) was used to screen and evaluate generalized anxiety symptoms with a total of seven items, using a scale from 0 (not at all) to 3 (almost every day) (21). The total score of the GAD-7 ranges from 0 to 21 points, where 0 to 5 points indicate no anxiety, and more than 5 points indicate anxiety states, and higher scores delineate higher anxiety severity. The Pittsburgh Sleep Quality Index (PSQI) is suitable for the assessment of sleep quality in healthy adults and patients with sleep disorders (22). The scale comprises 7 factors with 18 self-assessment questions, and each factor is scored on a scale of 0 to 3. The cumulative score of each factor component is the total score of the PSQI (0–21), the higher the score, the poorer the sleep quality. A PSQI score of 7 is often used as the cut-off value, and ≥7 points are defined as sleep disorders. The Morning and Evening Questionaire-5 (MEQ-5) was used to assess sleep rhythm status, with a total score ranging from 4 to 25 (23). The demarcation points were as follows: 4–11, 12–17, and 18–25 indicated night, intermediate, and morning types, respectively.

### Statistical analysis

In this study, all data were assessed and analyzed by an experienced statistician using SPSS 26.0. Two-sided *P* < 0.05 was considered statistically significant. Normal or skewed distributions of continuous variables were determined using the Kolmogorov–Smirnov test. Continuous variables were presented as mean ± standard deviation when they were normally distributed and as medians with interquartile ranges. Categorical variables were presented as numbers with percentages. The primary outcome was the incidence of re-positivity, and according to whether the reinfected patients were grouped into groups R and C. Student's *t*-tests or Mann–Whitney U tests were performed to compare the differences in continuous variables between the two groups. The chi-square test or Fisher's exact test was used to compare categorical variables between the two groups.

Univariate logistic regression was used to determine which factors had a statistically significant effect on re-positivity. These factors included infection status (asymptomatic or symptomatic), vaccination inoculation (yes or no), sex (male or female), age group (<60 or ≥60 years), BMI category (<18.5, 18.5–24 or >24), educational level (≤ 6, 6–12 or >12 years), occupation (professional, laborer, or other), smoking status (current smoker; former smoker, or never smoker), alcohol drinking status (current alcohol drinker, former drinker, or never drinker), health complications (yes or no), sleep status (PQSI ≤ 5 or PQSI > 6), MEQ-5 (morning, evening, or intermediate type), depression status (PHQ-9 <5 or PHQ-9 ≥ 5), and anxiety status (GAD-7 < 5 or GAD-7 ≥ 5). The criterion for inclusion in the regression equation was *P* < 0.1. Stepwise logistic regression analysis was then performed, and variables with *P* < 0.1 were included in the model. Odds ratios with 95% confidence intervals (CI) for significant factors of re-positivity were calculated. Additionally, time from infection to recovery, and time from recovery to re-positivity, were compared between different groups according to the identified significant factors, and Kaplan–Meier analysis was performed based on group allocation.

## Result

As shown in Figure 1, a total of 933 patients were screened, and 205 patients were excluded according to the exclusion criteria. Of the 728 included patients, 45 were excluded due to missing data or refusal to continue the study. Therefore, 683 patients were included in the final analysis. The demographic and preoperative data of all the included patients are presented in Table 1. Significant differences were found in the percentage of PQSI ≥ 6 (22.4 vs. 12.9%; *P* = 0.008) and PHQ-9 ≥ 5 (18.1 vs. 8.9%; *P* < 0.0001). There was no significant difference in other demographic or baseline data between the two groups.

**Table 1:** Demographic characteristics, data of psychological and sleep evaluation between patients in re-positivity and control patients.

	**Group R (*n* = 116)**	**Group C (*n* = 567)**	***P***
Age (year)	53.2 ± 16.9	50.5 ± 15.1	0.081
Sex (male/female)	48/68	206/361	0.305
Body max index (kg/m^2^)	23.2 ± 3.9	23.5 ± 3.9	0.353
Infection status (asymptomatic /symptomatic)	100/16	471/96	0.406
Vaccination inoculation (yes/no)	107/9	546/21	0.052
Educational level (≤ 6/6–12/>12)	13/52/51	67/236/264	0.816
Occupation (mental worker/manual worker/other)	27/47/42	142/271/154	0.138
Smoking status (current smokers/ former smokers/never smokers)	96/16/4	461/87/19	0.913
Alcohol drinking status (current alcohol drinker/former drinker/ never drinker)	99/15/2	467/86/14	0.720
Complication (yes/no)	28/88	109/458	0.229
Cardiovascular complication(yes/no)	15/101	51/516	0.191
Respiratory complication (yes/no)	4/112	16/551	0.715
Other systematic complication (yes/no)	12/104	44/523	0.355
Sleep status (PQSI ≥ 7/PQSI < 7)	26/96	73/494	0.008
MEQ-5 (morning type/evening type/intermediate type)	64/1/51	317/3/247	0.907
Depressed status (PHQ-9 ≥ 5/PHQ-9 < 5)	21/95	51/516	0.004
Anxiety status (GAD-7 ≥ 5/GAD-7 < 5)	17/99	50/517	0.054
Time from infection to recovery	16.9 ± 4.4	16.3 ± 4.4	0.166
Time from recovery to re-positivity	12(9–18)	/	/

PQSI, Pittsburgh Sleep Quality Index Scale; MEQ-5, Morning and Evening Questionaire-5; PHQ-9, Patient Health Questionnaire nine; GAD-7, Generalized Anxiety Disorder Scale seven; Complication means diseases of different systems (including the cardiovascular, respiratory, urinary, digestive, nervous, and hematological systems, etc.).

As shown in Table 2, in the univariate logistic regression of risk variables for predicting patients' re-positivity, age group (*P* = 0.005), sleep status (*P* = 0.009), and depressed status (*P* = 0.004) were identified as significant risk factors. In addition, vaccination inoculation (*P* = 0.058) and anxiety status (*P* = 0.057) were included in the overall logistic regression model. In the final overall logistic regression model, age group (*P* = 0.006) and depressed status (*P* = 0.006) were identified as independent risk factors for re-positivity. The odds ratios of age≥60 years and PHQ-9≥5 were 1.82 (95%CI:1.18–2.78) and 2.22 (1.27–3.85) respectively, for the prediction of re-positivity (Table 3).

**Table 2:** Univariate logistic regression of risk variables for predicting re-positivity.

	**Chi-square**	***P***
Age group (< 60/≥60 years)	7.865	0.005
Sex (male/female)	1.048	0.306
BMI group (< 18.5/18.5–24/>24)	3.799	0.150
Infection status (asymptomatic/symptomatic)	0.689	0.406
Vaccination inoculation (yes/no)	3.604	0.058
Educational level (≤ 6/6–12/>12)	0.405	0.817
Occupation (mental worker/manual worker/other)	3.927	0.140
Smoking status (current smokers/ former smokers/never smokers)	0.181	0.913
Alcohol drinking status (current alcohol drinker/former drinker/ never drinker)	0.635	0.721
Complication (yes/no)	1.443	0.230
Sleep status (PQSI ≥ 7/PQSI < 7)	6.882	0.009
MEQ-5 (morning type/evening type/intermediate type)	0.192	0.909
Depressed status (PHQ-9 ≥ 5/PHQ-9 < 5)	8.130	0.004
Anxiety status (GAD-7 ≥ 5/GAD-7 < 5)	3.628	0.057

PQSI, Pittsburgh Sleep Quality Index Scale; MEQ-5, Morning and Evening Questionaire-5; PHQ-9, Patient Health Questionnaire nine; GAD-7, Generalized Anxiety Disorder Scale seven. Complication means diseases of different systems (including the cardiovascular, respiratory, urinary, digestive, nervous, and hematological systems, etc.).

**Table 3:** Overall logistic regression model based on significative factors for predicting re-positivity.

**Predictors**	**Chi-square**	***P*-value**	**Odds ratio (95%CI)**
Age group (< 60/≥60 years)	7.433	0.006	1.82 (1.18–2.78)
Depressed status (PHQ-9 < 5/PHQ-9 ≥ 5)	7.653	0.006	2.22 (1.27–3.85)

CI, confidence interval; PHQ-9, patient health questionnaire nine.

**Figure 2:**
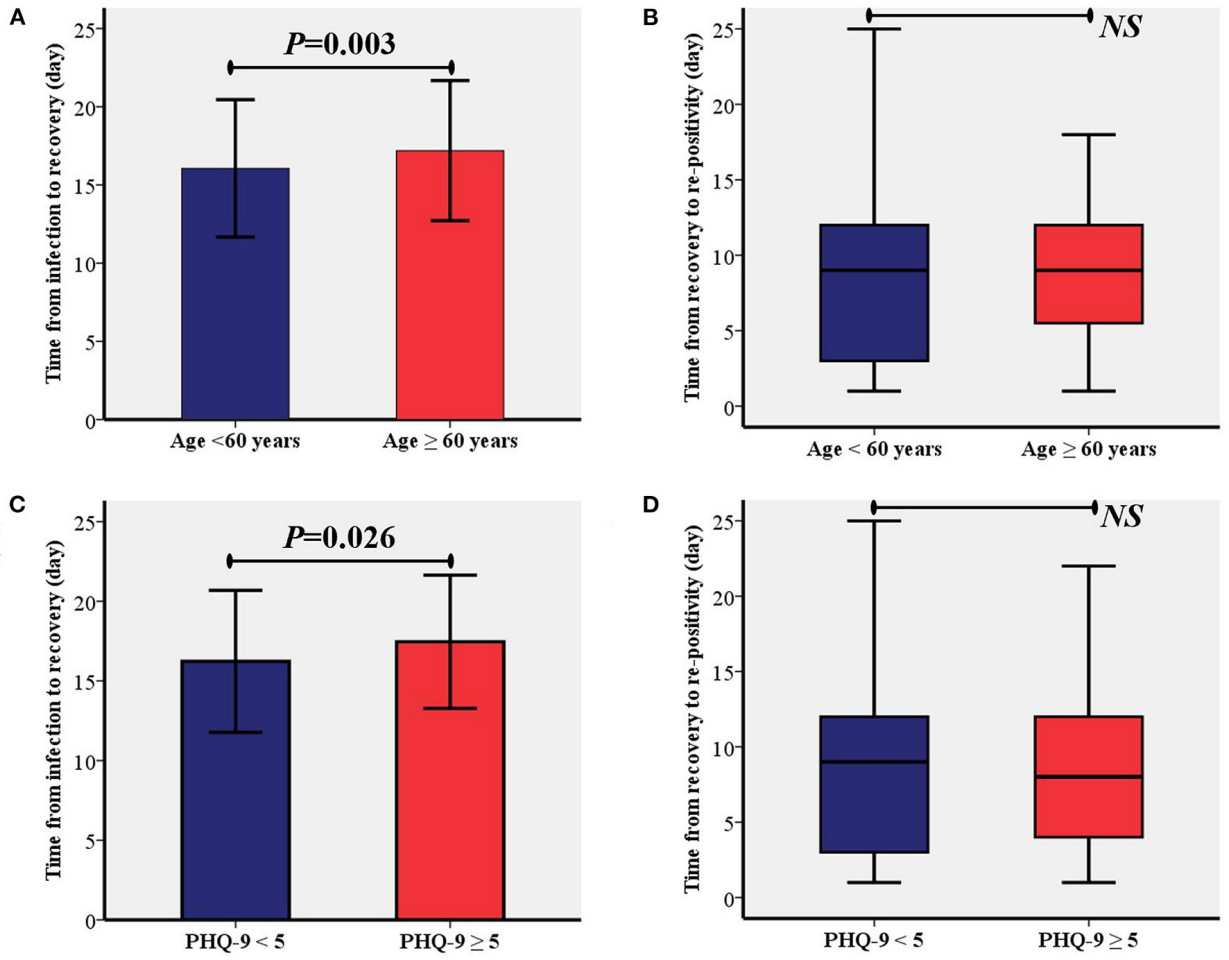
Comparisons of time from the infection to recovery and time from recovery to re-positivity between different age group **(A,B)** and depression status **(C,D)**. PHQ-9, patient health questionnaire nine; NS, Not Significant.

According to the two identified risk factors, time from infection to recovery and time from recovery to re-positivity between patients were compared between age of < 60 or ≥60 years and PHQ-9 < 5 or PHQ-9 ≥ 5, respectively. The comparative analysis showed that time from the infection to recovery in patients aged ≥60 years was significantly longer than in patients aged < 60 years (17.2 ± 4.5 *vs*. 16.0 ± 4.4, *P* = 0.003, Figure 2A). Moreover, the time from infection to recovery in patients with PHQ-9 ≥ 5 was significantly longer than in patients with PHQ-9 < 5 (17.5 ± 4.2 *vs*. 16.2 ± 4.5, *P* = 0.026, Figure 2C). As shown in Figures 2B,D, no significant differences were found between the different age groups or depressed groups.

**Figure 3:**
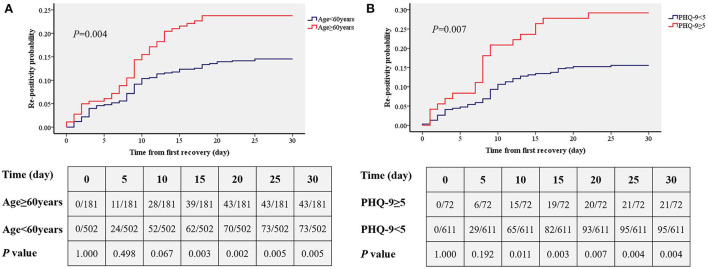
Comparisons of the accumulated rates of re-positivity between different age group **(A)** and depression status **(B)**. PHQ-9, patient health questionnaire nine.

Kaplan–Meier (Figures 3A,B) analysis showed that there was a significantly higher primary re-positivity rate in patients aged ≥60 years (*P* = 0.004) and PHQ-9≥5 (*P* = 0.007). On the 15^th^ day, patients with PHQ-9 ≥ 5 showed a significantly higher incidence of re-positivity compared to patients with PHQ-9 < 5 (21.5 vs. 12.4%, *P* = 0.003). On the 10^th^ day, patients aged ≥60 years showed a significantly higher incidence of re-positivity compared to patients aged < 60 years (20.1 vs. 10.6%, *P* = 0.011).

## Discussion

In this study, we found that of 683 Omicron-infected patients, there were 116 cases of re-positivity within 30 days after discharge from the hospital, and the overall re-positivity rate was 16.4%. Older adults over 60 years old and patients with depression were at risk for re-positivity. The time from infection to recovery was also longer in older adults and patients with depression. In addition, from the cumulative incidence curve analysis, we found that adults over 60 years of age showed a significant increase in re-positivity on the 15^th^ day, while re-positivity in depressed patients increased significantly on day 10.

Previous studies have demonstrated that reinfection with Omicron BA.2 can occur within 60 days, especially in unvaccinated individuals (24). In this study, the variant of Omicron was determined as BA.2. 2 by Sichuan Center for Disease Control and Prevention. And because after the outbreak the whole county was centralized controlled and all infected patients were isolated, thus we assumed that BA2.2 was the predominant version circulating in this region at this time. The re-positivity rate with Omicron BA.2.2 reached 16.4%, which is higher than that in a previous report (25). In addition, one study reported that reinfection was found in 26 (0.46%) of 5,554 alpha, 209 (1.16%) of 17,941 delta, and 520 (13.0%) of 3,992 Omicron variants (26). Thus, we speculated that reinfection might be more frequent in the Omicron BA.2.2 variant than in other variants.

Previous studies on Omicron reinfection have suggested a certain correlation between age and reinfection, and age >60 years is an important risk factor for reinfection (16). In this study, we found that the risk of re-positivity increased by nearly two times in patients aged over 60 years. Older adults often have more comorbidities; poor nutrition; and poor heart, lung, and kidney function, resulting in their low natural immunity and susceptibility to viruses (9). Although previous studies have suggested that Omicron has an immune evasion effect on vaccines or antibodies from previous infections (11–13), the infection and severe illness rates increase drastically when the patient is not vaccinated (8, 10). In this study, there was no statistically significant difference in vaccination status (35 unvaccinated persons) (*P* = 0.058), which may be related to the smaller sample size. This is attributed to government efforts, which vigorously promotes vaccination to reduce the number of unvaccinated people. Therefore, we recommend that the public be actively vaccinated to reduce the risk of re-positivity, especially in older adults. In addition, we found that the recovery time of older adults after infection with Omicron was longer than that of other patients, which further indicates that older adults are highly susceptible to Omicron infection. However, although it is statistically significant, the difference is relatively small and thus its clinical significance should be further assessed in future study.

In this study, depressive state was found to be an independent risk factor for increased re-positivity rates; that is, patients in a depressed state were more likely to be reinfected with Omicron. The risk of re-positivity increased by more than 2 times if the patient had a depressive status. This phenomenon may be associated with various physical and mental symptoms caused by depression, including decreased immunity, sleep disturbances, and so forth (27–29). In the univariate regression analysis of this study, sleep status was associated with re-positivity; however, sleep status was no longer an independent risk factor in multiple regression. This may be related to the effect of depression on sleep (27), and suggests that sleep problems caused by depressive states may also be involved in the increased risk of re-positivity. In addition, although anxiety status was not a significant factor for re-positivity in this study, it approached a statistically significant difference (*P* = 0.057). This may be related to the small sample size, and it is unclear whether it is related to re-positivity based on the current study. Depression may lead to decreased immunity, and immune escape by Omicron is common (13). Thus, patients may be more prone to re-positivity, which also explains why once the patient is infected with Omicron, it is difficult to fight Omicron through their own immune function. Longer monitoring and even more medical intervention may be required to increase the immune function of patients against re-positivity (30). In addition, it would be interesting to further analyze whether infected patients are in a depressed state. Specifically, in patients with depression, the time from infection to testing negative is also significantly longer than in patients without depression. This suggests that depression not only increases the re-positivity rate, but also prolongs the recovery time.

In addition to the risk factor regression analysis, this study also conducted a time-cumulative incidence analysis of different ages and different depression states, and the results showed that there was a significantly increased risk of re-positivity within 10 days for patients with depression. This was shorter than the effect of age (significant difference appeared at 15 days), suggesting that depressive status may be more influential than age. This study showed that depressive status and age >60 years are risk factors for re-positivity with Omicron. These findings are helpful to predict the risk of re-positivity, as well as for the prevention and diagnosis of Omicron. In addition, although patients with known mental disease were excluded, we speculated that it was also more vulnerable of re-positivity for those with diagnosed depression. Our findings are of great significance to implement control strategies, such as isolation, the management and control of close contacts and sub-close contacts, and the policy implementation of returning to society after discharge from the hospital after nucleic acid turns negative.

Several limitations of this study should be considered when interpreting its results. First, although vaccination status, anxiety, and sleep status were not identified as significant independent risk factors for re-positivity in this study, due to the high vaccination rate and relatively small sample size, their contribution to re-positivity cannot be completely excluded. Second, this study selected 30 days after recovery as the endpoint of the study. Although our investigators did not include patients with re-positivity after more than 25 days, it is necessary to extend the observation time in subsequent studies to further observe the impact of these related factors. Additionally, the psychological scales used in this study were mainly self-rating scales, and it is necessary to use more professional scales in future research to evaluate the effects of depression and other psychological states on Omicron re-positivity.

In summary, this study confirms that patients in a depressed state and older adults are at risk for re-positivity with Omicron, and depression and older age can prolong the time from infection to recovery. Therefore, in patients infected with Omicron, more attention should be paid to older adults and patients in a depressive state.

## Data availability statement

The original contributions presented in the study are included in the article/supplementary material, further inquiries can be directed to the corresponding author.

## Ethics statement

The studies involving human participants were reviewed and approved by People‘s Hospital of Linshui County, Guangan, China. The patients/participants provided their written informed consent to participate in this study. Written informed consent was obtained from the individual(s) for the publication of any potentially identifiable images or data included in this article.

## Author contributions

HP, ML, and SZ have jointly designed the research question, prepared the manuscript, and revised it. All authors contributed to the article and approved the submitted version.

## Funding

This study was supported by the Kuanren Talents Program of the Second Affiliated Hospital of Chongqing Medical University and Guangan Municipal Science and Technology Innovation Project (2019SYF11).

## Conflict of interest

The authors declare that the research was conducted in the absence of any commercial or financial relationships that could be construed as a potential conflict of interest.

## Publisher's note

All claims expressed in this article are solely those of the authors and do not necessarily represent those of their affiliated organizations, or those of the publisher, the editors and the reviewers. Any product that may be evaluated in this article, or claim that may be made by its manufacturer, is not guaranteed or endorsed by the publisher.

## References

[1] Parums DV. Editorial: The 2022 world health organization (WHO) priority recommendations and response to the Omicron variant (B.1.1.529) of SARS-CoV-2. Med Sci Monit. (2022) 28:e936199. 10.12659/MSM.936199 35102132 PMC8817616

[2] Ribeiro Xavier C Sachetto Oliveira R da Fonseca Vieira V Lobosco M Weber Dos Santos R. Characterisation of Omicron variant during COVID-19 pandemic and the impact of vaccination, transmission rate, mortality, and reinfection in South Africa, Germany, and Brazil. BioTech. (2022). 11:12. 10.3390/biotech11020012 35822785 PMC9264399

[3] Viana R Moyo S Amoako DG Tegally H Scheepers C Althaus CL. Rapid epidemic expansion of the SARS-CoV-2 Omicron variant in southern Africa. Nature. (2022) 603:679–86. 10.1038/s41586-022-04411-y 35042229 PMC8942855

[4] Manica M De Bellis A Guzzetta G Mancuso P Vicentini M Venturelli F. Intrinsic generation time of the SARS-CoV-2 Omicron variant: an observational study of household transmission. Lancet Reg Health Eur. (2022) 19:100446. 10.1016/j.lanepe.2022.100446 35791373 PMC9246701

[5] Kupferschmidt K Vogel G. How bad is Omicron? some clues are emerging. Science. (2021). 374:1304–5. 10.1126/science.acx9782 34882443

[6] Abdullah F Myers J Basu D Tintinger G Ueckermann V Mathebula M. Decreased severity of disease during the first global omicron variant covid-19 outbreak in a large hospital in Tshwane, South Africa. Int J Infect Dis. (2022) 116:38–42. 10.1016/j.ijid.2021.12.357 34971823 PMC8713416

[7] Madhi SA Kwatra G Myers JE Jassat W Dhar N Mukendi CK. Population immunity and Covid-19 severity with omicron variant in South Africa. N Engl J Med. (2022) 386:1314–26. 10.1056/NEJMoa2119658 35196424 PMC8908853

[8] Lu G Zhang Y Zhang H Ai J He L Yuan X. Geriatric risk and protective factors for serious COVID-19 outcomes among older adults in Shanghai Omicron wave. Emerg Microbes Infect. (2022) 11:2045–54. 10.1080/22221751.2022.2109517 35924388 PMC9448390

[9] Farheen S Agrawal S Zubair S Agrawal A Jamal F Altaf I. Patho-physiology of aging and immune-senescence: possible correlates with comorbidity and mortality in middle-aged and old COVID-19 patients. Front Aging. (2021) 2:748591. 10.3389/fragi.2021.748591 35822018 PMC9261314

[10] Iuliano AD Brunkard JM Boehmer TK Peterson E Adjei S Binder AM. Trends in disease severity and health care utilization during the early omicron variant period compared with previous SARS-CoV-2 high transmission periods - United States, December 2020-January 2022. MMWR Morb Mortal Wkly Rep. (2022) 71:146–52. 10.15585/mmwr.mm7104e4 35085225 PMC9351529

[11] Mohsin M Mahmud S. Omicron SARS-CoV-2 variant of concern: a review on its transmissibility, immune evasion, reinfection, and severity. Medicine. (2022) 101:e29165. 10.1097/MD.0000000000029165 35583528 PMC9276130

[12] Mohapatra RK Tiwari R Sarangi AK Islam MR Chakraborty C Dhama K. Omicron (B11529) variant of SARS-CoV-2: concerns, challenges, and recent updates. J Med Virol. (2022) 94:2336–42. 10.1002/jmv.27633 35118666 PMC9015506

[13] Bazargan M Elahi R Esmaeilzadeh A. OMICRON: virology, immunopathogenesis, and laboratory diagnosis. J Gene Med. (2022) 24:e3435. 10.1002/jgm.3435 35726542 PMC9350010

[14] Helfand M Fiordalisi C Wiedrick J Ramsey KL Armstrong C Gean E. Risk for reinfection after SARS-CoV-2: a living, rapid review for American college of physicians practice points on the role of the antibody response in conferring immunity following SARS-CoV-2 infection. Ann Intern Med. (2022) 175:547–55. 10.7326/M21-4245 35073157 PMC8791447

[15] Nguyen NN Houhamdi L Hoang VT Stoupan D Fournier PE Raoult D. High rate of reinfection with the SARS-CoV-2 Omicron variant. J Infect. (2022) 85:174–211. 10.1016/j.jinf.2022.04.034 35472367 PMC9033627

[16] Sacco C Petrone D Del Manso M Mateo-Urdiales A Fabiani M Bressi M. Risk and protective factors for SARS-CoV-2 reinfections, surveillance data, Italy, August 2021 to March 2022. Euro Surveill. (2022) 27:2200372. 10.2807/1560-7917.ES.2022.27.20.2200372 35593164 PMC9121659

[17] Li D Liao X Ma Z Zhang L Dong J Zheng G. Clinical status of patients 1 year after hospital discharge following recovery from COVID-19: a prospective cohort study. Ann Intensive Care. (2022) 12:64. 10.1186/s13613-022-01034-4 35816225 PMC9272871

[18] Jung YH Ha EH Choe KW Lee S Jo DH Lee WJ. Persistent symptoms after acute COVID-19 infection in Omicron era. J Korean Med Sci. (2022) 37:e213. 10.3346/jkms.2022.37.e213 35818704 PMC9274102

[19] Zhou J Ghose B Wang R Wu R Li Z Huang R. Health perceptions and misconceptions regarding COVID-19 in China: online survey study. J Med Internet Res. (2020) 22:e21099. 10.2196/21099 33027037 PMC7641649

[20] Chen Y Zhou R Chen B Chen H Li Y Chen Z. Knowledge, perceived beliefs, and preventive behaviors related to COVID-19 among Chinese older adults: cross-sectional web-based survey. J Med Internet Res. (2020) 22:e23729. 10.2196/23729 33293262 PMC7781588

[21] Huang XJ Ma HY Wang XM Zhong J Sheng DF Xu MZ. Equating the PHQ-9 and GAD-7 to the HADS depression and anxiety subscales in patients with major depressive disorder. J Affect Disord. (2022) 311:327–35. 10.1016/j.jad.2022.05.079 35598748

[22] Zitser J Allen IE Falgàs N Le MM Neylan TC Kramer JH. Pittsburgh sleep quality index (PSQI) responses are modulated by total sleep time and wake after sleep onset in healthy older adults. PLoS ONE. (2022) 17:e0270095. 10.1371/journal.pone.0270095 35749529 PMC9232154

[23] Shi C Luo JM Xiao Y. The association of sleep quality and burnout among Chinese medical residents under standardized residency training in a tertiary hospital. Sleep Breath. (2022) 23:1–8. 10.1007/s11325-022-02621-2 35460049 PMC9033310

[24] Vera-Lise I Dominik E Elisabeth R Kerstin H Raffael F Angelika X. Rapid reinfections with different or same Omicron SARS-CoV-2 sub-variants. J Infect. (2022) 85:e96–8. 10.1016/j.jinf.2022.07.003 35810939 PMC9262643

[25] Flacco ME Acuti Martellucci C Baccolini V De Vito C Renzi E Villari P. Risk of reinfection and disease after SARS-CoV-2 primary infection: meta-analysis. Eur J Clin Invest. (2022) 52:e13845. 10.1111/eci.13845 35904405 PMC9353414

[26] Özüdogru O Bahçe YG Acer Ö. SARS CoV-2 reinfection rate is higher in the Omicron variant than in the Alpha and Delta variants. Ir J Med Sci. (2022) 17:1–6. 10.1007/s11845-022-03060-4 35711013 PMC9203229

[27] Abel KM Carr MJ Ashcroft DM Chalder T Chew-Graham CA Hope H. Association of SARS-CoV-2 infection with psychological distress, psychotropic prescribing, fatigue, and sleep problems among UK primary care patients. JAMA Netw Open. (2021) 4:e2134803. 10.1001/jamanetworkopen.2021.34803 34783824 PMC8596199

[28] Abel KM Carr MJ Ashcroft DM Chalder T Chew-Graham CA Hope H. Relationship between anxiety, depression, and susceptibility to severe acute respiratory syndrome coronavirus 2 infection: proof of concept. J Infect Dis. (2022) 225:2137–41. 10.1093/infdis/jiac006 35022740 PMC8807218

[29] Wang S Quan L Ding M Kang JH Koenen KC Kubzansky LD. Depression, worry, and loneliness are associated with subsequent risk of hospitalization for COVID-19: a prospective study. Psychol Med. (2022) 19:1–10. 10.1017/S0033291722000691 35586906 PMC9924056

[30] Chekol Abebe E Tiruneh G Medhin M Behaile T Mariam A Asmamaw Dejenie T. Mutational pattern, impacts and potential preventive strategies of Omicron SARS-CoV-2 variant infection. Infect Drug Resist. (2022) 15:1871–87. 10.2147/IDR.S360103 35450114 PMC9017707

